# Taking the Pandemic by Its Horns: Using Work-Related Task Conflict to Transform Perceived Pandemic Threats Into Creativity

**DOI:** 10.1177/0021886320979649

**Published:** 2021-03

**Authors:** Dirk De Clercq, Renato Pereira

**Affiliations:** 1 Brock University, St. Catharines, Ontario, Canada; 2 Iscte Instituto Universitário de Lisboa, Avenida das Forças Armadas, Lisbon, Portugal; 3 ISCIM, Maputo, Mozambique

**Keywords:** perceived pandemic threats, work-related task conflict, creativity, collectivistic orientation, conservation of resources theory

## Abstract

This study investigates a pressing topic, related to the connection between employees’ perceptions that the COVID-19 pandemic represents a pertinent threat for their organization on one hand, and their exhibited creativity, a critical behavior through which they can change and improve the organizational status quo, on the other. This connection may depend on their work-related task conflict, or the extent to which they reach out to colleagues to discuss different perspectives on work-related issues, as well as their collectivistic orientation. Data were gathered from employees working in the real estate sector. The results inform organizational practitioners that they should leverage productive task conflict to channel work-related hardships, such as those created by the coronavirus pandemic, into creative work outcomes. This beneficial process may be particularly effective for firms that employ people who embrace collectivistic norms, so they prioritize the well-being of others.

## Introduction

Experiencing adverse, resource-depleting work situations can frustrate employees. It undermines the quality of their personal well-being, their professional functioning, and even their future career prospects (Ilies et al., 2020; Liao et al., 2019). In many cases, this sort of adversity results from the organizational context itself; when they get to work, employees might confront dysfunctional organizational politics (Jam et al., 2017), bullying behaviors by coworkers (Magee et al., 2017), or excessive workloads (Avery et al., 2010), for example. But adversity at work also spills over from private spheres. For example, family demands (Anand et al., 2015), rude treatment by family members (Lim & Tai, 2014), or experiences of life-threatening events (Raja et al., 2020) occur outside the work realm but have notable influences on work functioning. Another such external influence is highly salient today, in the form of the life-threatening COVID-19 pandemic (Ahorsu et al., 2020). The pandemic provokes extreme uncertainty and substantial fears among people who have to keep dealing with its effects on their health, their daily routines, their jobs, and their families (Hite & McDonald, 2020; Hokyu & Höllerer, 2020; Snell, 2020; Swaminathan & Mishra, 2020). How do employees experience and respond to this unprecedented, terrible situation, and what can organizations do to help them?

To answer these real-world challenges, this study investigates a specific behavioral response to perceived pandemic threats—defined as the extent to which employees ruminate on the negative organizational impact of the coronavirus—namely, engaging in productive idea clashes with organizational colleagues (Hoever et al., 2012; Jungst & Janssens, 2020). Work-related hardships due to the pandemic are deeply upsetting, so employees might try to take the proverbial bull by the horns and seek out ways to deal with it, such as by reaching out to colleagues and collectively engaging in work-related task conflict (Chen & Chang, 2005). In this sense, this article takes a unique perspective. Life-threatening events, such as terrorism or the pandemic, clearly can hinder employees’ ability to engage in productive work behaviors (e.g., Bader & Berg, 2014; De Clercq, Haq, & Azeem, 2017; Raja et al., 2020), and conflict often can be detrimental. But perceived threats also might encourage people to seek out productive forms of conflict. If they do, that productive conflict might have positive influences on employee creativity, which represents a critical change-oriented behavior that can improve the organizational status quo. For today’s employees, worried about the negative effects of the COVID-19 pandemic, we show that they can find a way to exhibit creativity by responding to the resource-draining threat with discretionary, productive task conflict. They should exchange divergent views with their peers about how to best address the hardships (Hobfoll & Shirom, 2000).

We also anticipate that the benefits of work-related task conflict are particularly prominent among employees with strong collectivistic orientations (Wang et al., 2017). These employees consider themselves part of a collective and assign greater weight to group rather than personal interests (Astakhova, 2015; Triandis, 2001), so the detrimental impact of the pandemic on the organizational collective may be especially worrisome for them (Dirani et al., 2020; Hite & McDonald, 2020). Employees who prioritize group interests should feel particularly motivated to resolve pandemic-related work challenges and enter into intensive exchanges of conflicting viewpoints with colleagues, in the hope that such efforts will help everyone cope with those threats (Moorman & Blakely, 1995; van Dyne et al., 2000). Through their enhanced willingness to engage in work-related task conflict, they might be especially well positioned to generate novel, pertinent solutions (De Clercq, Rahman, & Belausteguigoitia, 2017), a capacity that managers should acknowledge and build on for the good of the collective.

### Conservation of Resources Theory

To ground our somewhat unconventional, theoretical arguments about the beneficial role of employees’ perception of pandemic threats in spurring creativity, we draw on conservation of resources (COR) theory (Hobfoll et al., 2018). This theory stipulates that employees’ work behaviors are influenced powerfully by their motivation to counter any experienced resource losses with resource gains (Hobfoll & Shirom, 2000). For example, resource-draining work situations direct employees to undertake work activities that enable them to overcome or undo the resource drainage (De Clercq & Belausteguigoitia, 2017). We regard worrying about the negative organizational impacts of the pandemic as a significant drain on their resources (Caligiuri et al., 2020). To counter it, they might reach out to organizational colleagues to discuss ways to address the precarious situation (Jehn & Mannix, 2001). By initiating conflict-laden conversations, employees can mitigate their resource-depleting fears about the coronavirus and its harms (Hite & McDonald, 2020). In turn, they expend energy to gain more creative solutions for improving the organizational status quo (Zhang & Zhou, 2019), which is a type of resource.

Furthermore, COR theory posits that personal factors invigorate this process. For example, resource gains might result from the personal satisfaction that employees feel when they can formulate adequate behavioral responses to problems (Hobfoll et al., 2018; Ryan & Deci, 2000). We specifically propose that employees’ collectivistic orientation (Wang et al., 2017) may increase the likelihood that they see pandemic fears as opportunities. It inspires them to engage with other organizational members and compare ideas about how to move forward and protect the shared organization. A collectivistic orientation could motivate employees to share pertinent opinions, even if it risks raising task conflict, because their ultimate goal is to help the overall collective reduce work-related hardships caused by the pandemic (Lofquist & Matthiesen, 2018; Yang, 2019). By testing whether a collectivistic orientation invigorates the link between perceived pandemic threats and work-related task conflict, we pinpoint a relevant, personal boundary condition that can stimulate productive behaviors by collectivistic employees, even when a life-threatening virus infuses significant uncertainty in their work setting (Hite & McDonald, 2020).

### Relevance of the Study

This research offers recommendations for organizations negatively affected by the COVID-19 pandemic. It also suggests insights into other types of crises and employees’ responses to them (Schwarz & Stensaker, 2020). First, it details *how* employees’ beliefs about the coronavirus’s impact can stimulate their creativity. It occurs through their dedicated efforts to involve colleagues in productive discussions and clashes, in efforts to protect the organization from external threats (Jehn et al., 1999). Employees’ propensity to connect and exchange potentially conflicting work-related viewpoints with colleagues is an unexplored behavioral mechanism through which a negative environmental situation can produce benefits, such as creative output. This approach complements and proposes a new perspective on research that notes how factors such as insomnia (Toker et al., 2015) or job-related anxiety (De Clercq, Haq, & Azeem, 2017) might mediate the link between worries about life-threatening events (e.g., terrorism) and *negative* work outcomes. The pandemic clearly has detrimental ramifications, but it also might prompt employees to exhibit greater creativity, because they feel motivated to overcome the resource drainage and confront adversity head on, in collaboration with their colleagues (Bai et al., 2016). For organizational practitioners seeking to maintain their operations during the COVID-19 era, this study thus offers seemingly unexpected insights. To deal with the life-threatening crisis, they should encourage productive, conflict-laden exchanges among employees (Farh et al., 2010). In particular, they should work to establish a corporate culture that encourages helpful debates over ideas, such as by establishing formal or informal training programs (Ahadi & Jacobs, 2017; Enos et al., 2003). Those exchanges can improve the current organizational situation, through employees’ enhanced creativity, as long as managers take care to keep the conflicts productive and helpful to participants trying to cope with the pandemic.

Second, we pinpoint some conditions in which these links are more likely to materialize. A collectivistic orientation increases the perceived attractiveness of overcoming work-related hardships to aid everyone in the organization (Triandis, 2001). Extant research cites a *direct* positive impact of employees’ collectivistic orientations on positive work attitudes or activities, such as organizational commitment (Cohen & Keren, 2008), loyalty to the supervisor (Wang et al., 2017), or organizational citizenship behavior (Baeza & Wang, 2016). We propose a more nuanced and indirect, but still important, effect. Collectivism can generate a sense of personal accomplishment when employees exchange divergent viewpoints to come up with a plan for shielding their organization (Li et al., 2018; Ryan & Deci, 2000). If organizational practitioners want to *diminish* the risk that employees remain passive in the face of pandemic-induced organizational challenges, they should recruit and retain employees with strong collectivistic tendencies.

**Figure 1.**
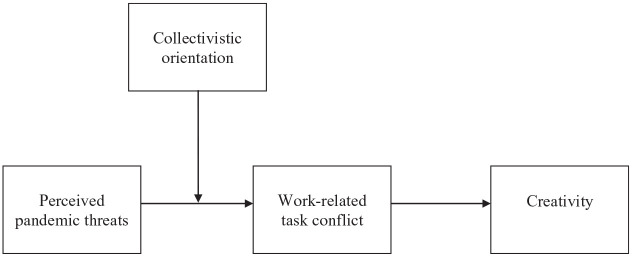
Conceptual model.

Figure 1 shows the proposed conceptual framework. With a grounding in COR theory (Hobfoll & Shirom, 2000), it proposes that resource-depleting perceptions about the threat of the COVID-19 pandemic lead to increased creativity levels, because employees seek to grab the bull by the horns and deal with the resource losses stemming from the perceived threat by engaging in content-related conflict. Their collectivistic orientation serves as a contingency factor. Worrying about the pandemic transforms into enhanced creativity, through work-related task conflict, particularly when employees grant more precedence to group than to individual interests.

## Hypotheses

### Mediating Role of Work-Related Task Conflict

The constant, prevalent threats associated with the COVID-19 pandemic make it likely that employees cannot stop thinking about it, which depletes their individual resource reservoirs (Ahorsu et al., 2020; Swaminathan & Mishra, 2020). In COR theory, employees then may dedicate individual energy to other work activities, reflecting their need or desire to *counter* the negative consequences of being exposed to resource-draining conditions (Hobfoll, 2001). When they worry about the dangers of the coronavirus, including how it might damage their firm or hinder their work functioning (Hite & McDonald, 2020), employees likely feel strong motives to *contain* the harmful situation. That goal might be possible if they debate productively with their colleagues about the best solutions (Jehn & Mannix, 2001). That is, concerns about the negative organizational impacts of the pandemic may stimulate people to reach out to other organizational members, initiate potentially heated discussions about how to handle the situation, and arrive at a consensus about viable solutions (Jehn & Bendersky, 2003; Zhang & Zhou, 2019). Conversely, if employees worry less about risks to their organization, they sense little threat to their own work situation and see no need to enter into difficult discussions about work issues (Swaminathan & Mishra, 2020).

**Hypothesis 1:** There is a positive relationship between employees’ perceived pandemic threats and their engagement in work-related task conflict.

Work-related task conflict generally increases creativity. When employees reach out to colleagues to exchange opinions about work-related issues, they gain better insights for developing novel solutions (Zhang & Zhou, 2019). Effective solutions require new combinations of varied and pertinent knowledge, which an individual employee cannot possess alone (Amason, 1996). Work-related task conflict provokes new insights into how to alter the organizational status quo (Chen & Chang, 2005; Lu et al., 2011). This logic is consistent with COR theory. Constructive exchanges of different viewpoints generate knowledge resources, which can generate additional resource gains in the form of creative work outcomes (De Clercq & Pereira, 2020; Hobfoll et al., 2018). Moreover, novel ideas tend to be controversial, even if they propose improvements, because change can be disruptive or undermine the current privileges that other members enjoy (Chen et al., 2015; Yuan & Woodman, 2010). Creative solutions that stem from exchanges of different opinions about work topics might avoid reactance though, because they reflect a range of opinions and identify potential criticisms in advance (Jehn & Bendersky, 2003). If people instead avoid productive work-related task conflict, they may be ill prepared to cope with skepticism or resistance to their novel ideas (van Dijk & van Dick, 2009). In that case, they may be less willing to undertake the effort needed to generate such ideas (De Clercq, Rahman, & Belausteguigoitia, 2017).

**Hypothesis 2:** There is a positive relationship between employees’ engagement in work-related task conflict and their creativity.

Combining Hypotheses 1 and 2 suggests a mediating role of work-related task conflict. It may translate employees’ perceived pandemic threats into enhanced creativity. The probability that employees who believe their organization is threatened by the pandemic go out of their way to develop new ideas increases if they are more likely to exchange work-related viewpoints with colleagues (Jehn & Mannix, 2001). Task conflict mediates other, similar links, such as when informational diversity (Jehn et al., 1999) or high-involvement work practices (Lee et al., 2015) indirectly produce beneficial work outcomes. We add the proposition that constructive conflict can serve as a valuable conduit between a negative situation (COVID-19 threats) and creativity too.

**Hypothesis 3:** Employees’ engagement in work-related task conflict mediates the relationship between perceived pandemic threats and creativity.

### Moderating Role of Collectivistic Orientation

Employees with a strong collectivistic orientation express concerns for the well-being of other people, rather than focusing on their individual interests (Moorman & Blakely, 1995; Wang et al., 2017). According to COR theory, employees direct their individual energy toward work activities that are consistent with their personal values, because consistency generates resource gains (e.g., personal satisfaction; Hobfoll et al., 2018). Therefore, collectivistic employees who perceive pandemic threats may be more likely to spark constructive peer exchanges in an attempt to benefit everyone by finding effective ways to diminish the negative impact of the crisis (Jehn & Bendersky, 2003). In contrast, employees without a collectivistic orientation tend not to worry much about their organization or colleagues (Triandis, 2001; van Dyne et al., 2000). They have less reason to leverage their pandemic fears constructively in task conflict efforts (Hobfoll & Shirom, 2000). They also experience less personal fulfillment from dedicating themselves to discretionary activities (Ryan & Deci, 2000). Therefore, they likely adopt passive approaches in their interactions with colleagues (Wang et al., 2017).

**Hypothesis 4:** The positive relationship between employees’ perceived pandemic threats and their engagement in work-related task conflict is moderated by their collectivistic orientation, such that this relationship is stronger among employees who are more collectivistic.

In tandem, the arguments for a catalytic role of collectivistic orientation and a mediating role of work-related task conflict indicate a moderated mediation dynamic (Preacher et al., 2007). Employees’ collectivistic orientation functions as a critical contingency in the indirect relationship between perceived pandemic threats and creativity, through work-related task conflict. Formally, devoted efforts to reach out to colleagues and confront varying opinions about work (Jehn et al., 1999) are behavioral mechanisms that underpin the connection between perceived pandemic threats and creativity levels, and they may be more salient among employees with a strong collectivistic orientation (Wang et al., 2017). Conversely, work-related task conflict should be a less instrumental factor for explaining how worries about the pandemic enhance the creativity of employees who are less concerned about others’ well-being.

**Hypothesis 5:** The indirect relationship between employees’ perceived pandemic threats and their creativity, through their engagement in work-related task conflict, is moderated by their collectivistic orientation, such that this indirect relationship is stronger among employees who are more collectivistic.

## Research Method

### Sample and Data Collection

The hypotheses were tested with a survey administered to a sample of employees of a real estate organization in Portugal. The survey items were translated from English into Portuguese, by two translators fluent in both languages who applied the translation–back-translation method (Brislin, 1986). The organization under study provides various property brokerage services, such as supporting real estate searches, promotion, and legal registry. Portuguese society is marked by high levels of collectivism (Hofstede et al., 2010), so these employees may be particularly likely to go out of their way to address the pandemic in ways that contribute to the well-being of the organization and its constituents.

In addition to providing a relevant study context in general, Portugal is appropriate for our research purposes in terms of its experiences with the coronavirus. The survey was administered in June 2020, a month when the COVID-19 pandemic was particularly severe in Portugal. The number of confirmed new cases had jumped by 29%, and the death toll had risen by 10%, compared with the previous 30-day period (Direção-Geral da Saúde, 2020). Furthermore, the country had been subjected to strict lockdown mandates in mid-March, some of which were being gradually lifted by June. But even as the restrictions eased, such that nonessential workers were allowed to return to their jobs around June 1 (Conselho de Ministros, 2020), many people continued to work from home and suffer significant threats to their well-being and livelihood.

With the cooperation of the real estate company, we sent surveys to 250 employees, randomly selected from an employee list provided by senior management. Cover letters guaranteed the complete confidentiality of all answers, noted that their participation was totally voluntary, and emphasized that they could withdraw at any time. We also highlighted that our research objective was to identify broad-stroke patterns in the composite data rather than any individual-level findings. Furthermore, the cover letters explicitly noted that there were no right or wrong answers and that responses to specific questions would likely differ across respondents. We thus asked for honest answers. These features help minimize social desirability biases. Of the 250 original surveys, we received 128 completed responses, for a response rate of 51%. Among these 128 respondents, 34% were women, and 53% had worked for their organization for more than 5 years.

### Measures

The study constructs were assessed with items drawn from previous studies. The measurement scales utilized 7-point Likert-type anchors that ranged between 1 = *strongly disagree* and 7 = *strongly agree*.

#### Perceived Pandemic Threats

To measure employees’ beliefs about the negative impact of the COVID-19 pandemic on their organization, we adapted a well-established 13-item scale of perceived threats of terrorism to a virus context (Raja et al., 2020; Sinclair & LoCicero, 2006). Three sample items were “I frequently find myself preoccupied with thinking about the impact of COVID19 on my organization,” “The threat that COVID-19 poses to my organization often enters my mind,” and “I worry that the threat of COVID-19 to my organization will never end” (Cronbach’s α = .84).

#### Work-Related Task Conflict

To evaluate the extent to which employees reach out to colleagues expressly for the purpose of involving them in the exchange of conflicting viewpoints about work, we used a four-item scale task conflict (De Clercq, Rahman, & Belausteguigoitia, 2017). In light of our theoretical focus on employees’ proactive task conflict engagement, we adapted the wording slightly, to capture whether employees actually initiate the conflict, such as by asking whether they agree that “I often reach out to colleagues to discuss different opinions about projects” and “I often reach out to colleagues to pinpoint conflicting viewpoints” (Cronbach’s α = .94).

#### Creativity

We assessed the extent to which employees generate novel ideas that improve the organizational status quo with a three-item scale of creative performance (Janssen, 2001). Participants rated, for example, whether “I often generate original solutions to organizational problems” and “I often create new ideas for organizational improvement” (Cronbach’s α = .87). A self-rated measure of creativity is common in prior research (e.g., Kühnel et al., 2020; Sijbom et al., 2018), as well as theoretically justified, because other organizational members, such as peers, rarely have a complete view of the range of creative behaviors that individual employees undertake (Elsbach & Kramer, 2003; Ford, 1996; Zhou et al., 2008).

#### Collectivistic Orientation

We assessed the extent to which employees prioritize others’ well-being, rather than focusing on their personal interests, with a four-item scale of collectivistic orientation (Triandis & Gelfand, 1998). They indicated, for example, whether “The well-being of my peers is important to me” and “If a peer gets a prize, I would feel proud” (Cronbach’s α = .94).

#### Control Variables

The statistical analyses accounted for the effects of two control variables: gender (1 = female) and organizational tenure (1 = *5 years or less*, 2 = *between 6 and 10 years*, 3 = *between 11 and 15 years*, 4 = *between 16 and 20 years*, 5 = *more than 20 years*). Women tend to avoid conflict more than men, so they might shy away from exchanging possibly controversial, conflicting viewpoints (Jehn & Bendersky, 2003), but they also exhibit a greater tendency to seek to enhance organizational well-being with productive work activities, such as creativity (Baer & Kaufman, 2008). Employees who have worked for the organization longer also might be more confident in the successful outcomes of their productive work behaviors, even if those behaviors are potentially disruptive (Gong et al., 2009).

### Statistical Technique

The Process macro approach (Hayes et al., 2017) provides the test of the study hypotheses, consistent with the methods applied by previous studies that also predict mediation or moderated mediation frameworks (e.g., Han et al., 2019; Tresi & Michelič, 2018). This approach does not assume the normality of (conditional) indirect effects and relies on a bootstrapping procedure (MacKinnon et al., 2004). To assess the mediation effect, we estimated the indirect relationship between perceived pandemic threats and creativity through work-related task conflict, along with the confidence interval (CI), by using the Process macro’s Model 4. This first step includes an assessment of the signs and significance levels of the direct paths between (1) perceived pandemic threats and work-related task conflict and (2) work-related task conflict and creativity. We check for the presence of moderated mediation by comparing the effect sizes in the conditional relationships when the moderator is one standard deviation (*SD*) below its mean, at its mean, and one *SD* above its mean. In line with the proposed theoretical framework, we ran the Process macro’s Model 7, to estimate the moderating effect of collectivistic orientation on the path between perceived pandemic threats and work-related task conflict but not between work-related task conflict and creativity.^1^

## Results

Table 1 reports the correlation coefficients and descriptive statistics, and Table 2 reports the mediation results obtained from the Process macro. Perceived pandemic threats increase work-related task conflict (β = .580, *p* < .001, Hypothesis 1), which spurs creativity (β = .512, *p* < .001, Hypothesis 2). The effect size of .297 for the indirect relationship between perceived pandemic threats and creativity through work-related task conflict and a CI that does not include 0 [.161, .472] provide evidence of the presence of mediation (Hypothesis 3).

**Table 1.** Correlation Table and Descriptive Statistics.

	1	2	3	4	5	6
1. Perceived pandemic threats						
2. Work-related task conflict	.609**					
3. Creativity	.443**	.548**				
4. Collectivistic orientation	.323**	.201*	.454**			
5. Gender (1 = female)	.094	−.078	−.193*	−.106		
6. Organizational tenure	.017	−.057	.092	−.073	−.238**	
*M*	3.125	2.701	4.505	5.088	.336	1.828
*SD*	1.381	1.258	1.497	1.135	.474	.879

*Note. N* = 128.

* *p* < .05. **p < .01.

**Table 2.** Mediation Results (Process macro).

	Work-related task conflict		Creativity	
Gender (1 = female)	−.443*		−.325	
Organizational tenure	−.158		.198†	
Perceived pandemic threats	.580***		.085	
Collectivistic orientation	−.034		.448***	
Work-related task conflict			.512***	
*R* ^2^	.401		.456	
	Effect size	Bootstrap *SE*	LLCI	ULCI
Indirect effect	.297	.080	.161	.472

*Note. n* = 128; LLCI = lower limit confidence interval; UCLI = upper limit confidence interval.

† *p* < .10. *p < .05. **p < .01. ***p < .001.

The findings in Table 3 also indicate a positive, significant effect of the perceived pandemic threats × collectivistic orientation interaction term (β = .140, *p* < .01, Hypothesis 4) in predicting work-related task conflict. In particular, the Process macro findings reveal that the relationship between perceived pandemic threats and work-related task conflict is stronger at higher levels of collectivistic orientation (.434 at one *SD* below the mean, .609 at the mean, and .748 at one *SD* above the mean). We test for the presence of moderated mediation (Hypothesis 5) by comparing the strength of the conditional indirect relationship between perceived pandemic threats and creativity (through work-related task conflict) at different levels of collectivistic orientation. Table 3 reveals the increasing effect sizes at higher levels of the moderator: from .143 at one *SD* below the mean, to .200 at the mean, to .246 at one *SD* above the mean. With a formal test of moderated mediation, which assesses the index of moderated mediation and its corresponding CI (Hayes, 2015), we find that the index equals .046, and its CI does not include 0 [.019, .090]. Thus, a collectivistic orientation invigorates the positive indirect relationship between perceived pandemic threats and creativity, through work-related task conflict, consistent with Hypothesis 5 and the overall proposed conceptual framework.

**Table 3.** Moderated Mediation Results (Process Macro).

	Work-related task conflict		Creativity	
Gender (1 = female)	−.407*		.591***	
Organizational tenure	−.149		−.024	
Perceived pandemic threats	.610***		–.178**	
Collectivistic orientation	−.030		.861***	
Perceived pandemic threats × Collectivistic orientation	.140**			
Work-related task conflict			.329***	
*R* ^2^	.435		.727	
Conditional *direct* effect of perceived pandemic threats on work-related task conflict				
	Effect size	Bootstrap *SE*	LLCI	ULCI
−1 *SD*	.434	.086	.264	.603
*M*	.609	.067	.475	.742
+1 *SD*	.748	.091	.568	.929
Conditional *indirect* effect of perceived pandemic threats on creativity				
	Effect size	Bootstrap *SE*	LLCI	ULCI
−1 *SD*	.143	.044	.063	.236
*M*	.200	.049	.113	.306
+1 *SD*	.246	.059	.140	.375
Index of moderated mediation	.046	.018	.019	.090

*Note. n* = 128; LLCI = lower limit confidence interval; UCLI = upper limit confidence interval.

* *p* < .05. **p < .01. ***p < .001.

## Discussion

This research is motivated by an important and timely topic: How does the COVID-19 crisis interfere with organizational life, and how do employees respond in the course of their work? We examine the link between perceived pandemic threats and creativity levels, with specific attention to two factors that help explain this link. Consistent with the logic of COR theory (Hobfoll et al., 2018), we theorize that (1) dedicated efforts to engage other organizational members in productive idea clashes lead employees’ worries about the coronavirus to transform into an enhanced propensity to generate novel ideas for organizational improvement, and (2) this mediating role of work-related task conflict is more pronounced among employees with a strong collectivistic orientation. The empirical results provide support for our theoretical predictions; they also offer insights beyond the specific effects of the coronavirus.

Extant research normally focuses on the harmful effects of life-threatening events (Kastenmüller et al., 2014; Raja et al., 2020; Toker et al., 2015). But as we show, employee concerns about how their organization is being harmed also can stimulate them to address the problem, such that they willingly enter into constructive idea clashes with their organizational peers (Jehn & Mannix, 2001; Jungst & Janssens, 2020). Employees’ concern that their organization might suffer significant damage due to external events, such as a global virus, stimulates them to take the pandemic by the horns and devote significant energy to share their views. Then together, they can find the best way forward (Quinn et al., 2012). These actions reflect employees’ desire to limit the negative work consequences of the pandemic and enhance the internal functioning of the organization. They likely produce novel solutions along these lines (De Clercq, Rahman, & Belausteguigoitia, 2017). In COR terms, employees seek to reduce work-related resource losses by engaging in constructive opinion clashes and creative behaviors, in an attempt to ensure resource-generating outcomes (Hobfoll & Shirom, 2000).

This mediating effect of work-related task conflict is reinforced by collectivistic orientations, because people with such world views gain additional resources, such as joy and satisfaction, from engaging with colleagues in productive idea clashes (Lofquist & Matthiesen, 2018; Moorman & Blakely, 1995). They anticipate more value of engaging in work-related task conflict, because their pertinent personal factors make this response to resource-draining situations more attractive (Hobfoll & Shirom, 2000). Collectivistic employees derive personal satisfaction from transforming a negative situation into a positive outcome for the collective (van Dyne et al., 2000). Therefore, they feel motivated to address work-related hardships by collaborating with colleagues, even if doing so creates some conflict (Triandis, 2001; Wang et al., 2017). In essence, they feel fulfilled by their active initiation of such discussions during the pandemic (Ryan & Deci, 2000). In contrast, employees who are less collectivistic and more focused on their individual well-being do not feel compelled to help the organization or its members. They are unlikely to respond to pandemic threats by trying to bring different viewpoints and solutions into the open. Their subsequent creativity then tends to be lower (Jehn & Bendersky, 2003; Yang, 2019).

Taken together, these results offer valuable insights into *how* and *when* employees’ preoccupations with the COVID-19 pandemic and its impacts might enhance their work behaviors. First, employees’ active participation in work-related disagreements is an important mechanism that links beliefs about the detrimental impacts of the pandemic for organizational functioning, as a work hardship, with increased creativity levels. Second, concerns about collective well-being, instead of personal interests, fuel this process.

### Limitations and Future Research

This study has some limitations; they offer opportunities for continued research. First, some care is required with regard to the presence of reverse causality. For example, creative behaviors could increase employees’ awareness of the different skills that their colleagues possess, which might influence their perceptions about pandemic-evoked work challenges. Measuring these constructs over time could provide a formal test of their causality. Still, our conceptual framework reflects the widely used COR framework, in which employees react to resource-draining circumstances with activities to avoid additional resource losses (Hobfoll & Shirom, 2000). Further measures also could specify whether employees’ propensity to engage in work-related task conflict, in response to perceived pandemic threats, really is guided by their motivation to help the organization and their work functioning.

Second, as must any parsimonious study, we ignored alternative possible mechanisms. Our focus on task conflict acknowledges arguments that it can transform resource-draining situations into positive outcomes (Hobfoll et al., 2018). Other behavioral responses could be similarly effective, such as problem-oriented voice (Liang et al., 2012) or idea championing (Walter et al., 2011). Beliefs and attitudes can be influential too, whether involving employees’ sense of responsibility for their organization’s well-being (Lin & Tsai, 2020) or fear of losing their jobs (De Spiegelaere et al., 2014), for example. A useful extension then might compare the explanatory power of work-related task conflict with that of these alternative mechanisms, or else test potential sequential mediation models (e.g., pandemic threats spur creativity first through perceived job insecurity and then through work-related task conflict).

Third, a collectivistic orientation is highly relevant for this study. It aligns closely with the idea that collectivist employees feel motivated to react to crises with resource-gaining work efforts to help the organizational collective. But continued research could consider other relevant personal resources, such as resilience (Al-Hawari et al., 2020) or passion for work (Klaukien et al., 2013), that might mitigate the hardships of external crises. Such influences are not limited to personal resources either. Pertinent *contextual* factors could catalyze the relationship between perceived pandemic threats and work-related task conflict, including perceived person–organization fit (Ruiz-Palomino & Martínez-Cañas, 2014) or psychological contract fulfillment (Choi et al., 2019). Researchers might investigate the relative potency of various moderators, particularly in comparison with the invigorating role of collectivistic orientation.

Fourth, our conceptual arguments are not country-specific, and we expect that the nature of the hypothesized relationships should apply broadly, even if the *strength* of the relationships might vary with pertinent cultural factors. Notably, Portugal scores high on both uncertainty avoidance and collectivism (Hofstede et al., 2010). Employees in our study context thus might feel particularly upset by the uncertainty of the pandemic and react vigorously to undo the associated hardships. Their desire for group harmony and prioritization of common interests also might mean that employees’ individual-level collectivism has a particularly powerful effect, in terms of leveraging perceived pandemic threats into enhanced work-related task conflict and creativity. Cross-country *comparisons* should explicitly examine the potential effects of cultural features in the proposed conceptual framework.

### Practical Implications

Most employees today sense some threat related to the COVID-19 pandemic, prompting significant preoccupation about their current and future work functioning, as well as negative implications for their professional well-being (Hite & McDonald, 2020). We identify a possible positive outcome of such preoccupations too. To the extent that employees worry about their organization, they may allocate energy resources to exchanges of different viewpoints with colleagues. Those exchanges then can have beneficial consequences for their creativity. When pursuing such positive outcomes, managers need to take care to limit persistent disagreements about content-related issues though. Continual in-fighting can escalate into negative relationship dynamics (Simons & Peterson, 2000). For organizational practitioners, we offer an explicit recommendation: Create an internal culture that encourages and accepts idea clashes, but only as long as they are productive and help the participants cope with external threats to their work functioning. Open sharing forums should be designed to mandate respectful behaviors and to demonstrate helpful forms of conflict. Clear organizational communication should describe how such interactions can help address the challenges of a global pandemic crisis (Sanders et al., 2020). These interactions could have other positive impacts too, such as helping employees gain a sense of belonging or shared interests. In turn, they might come to agree in their desire to exchange different viewpoints and generate novel solutions to the crisis (Cabrera & Cabrera, 2005).

Some employees may be especially well positioned to engage with colleagues in this productive work-related task conflict, whether during a pandemic or in general. When employees genuinely seek collective instead of personal interests (Wang et al., 2017), they are better able to address and mitigate work-related hardships. In addition to making collectivistic views part of the corporate culture, organizations should recognize that some employees will experience personal satisfaction from protecting the organization or helping their colleagues succeed. Then managers should use these insights to optimize *assignments* to specific job tasks and projects, including those that involve increased exposure to the coronavirus. For example, outside sales representatives must meet with clients, so they suffer more exposure to these dangers than do back-office support staff who can work completely from home. Arguably, collectivistic employees in jobs that involve greater pandemic threats may be particularly dedicated to combining knowledge throughout the organization, to come up with creative solutions to minimize their own and others’ risks. Such assignments ultimately might increase the chances that the organization overall finds creative solutions to the work-related harms inflicted by the pandemic.

## Conclusion

This study has sought to advance understanding of the interplay between the current COVID-19 crisis and an organization’s internal functioning, though the implications can speak to other types of crises and difficult work conditions too. Employees’ worries about the negative organizational impact of the crisis can trigger creativity, to the extent that they seek to overcome the resource-draining situation by pursuing constructive conflict and integrating different viewpoints to craft pertinent solutions. This beneficial role of work-related disagreements appears contingent on employees’ desire to help their organizational peers with these efforts. Thus, this role is more powerful among collectivistic employees. As the COVID-19 crisis persists, further studies should use our findings as a springboard and pursue additional insights into how employees’ dedicated work efforts might transform external threats to organizational well-being into beneficial outcomes, in combination with the influence of valuable individual features of employees.

## Notes

1. A follow-up analysis confirmed that a collectivistic orientation did not significantly moderate this second path.
